# Nanomedicine for Imaging and Therapy of Pancreatic Adenocarcinoma

**DOI:** 10.3389/fbioe.2019.00307

**Published:** 2019-11-13

**Authors:** Giulia Brachi, Federico Bussolino, Gianluca Ciardelli, Clara Mattu

**Affiliations:** ^1^Politecnico di Torino, DIMEAS, Turin, Italy; ^2^Department of Oncology, University of Torino, Turin, Italy; ^3^Candiolo Cancer Institute -IRCCS-FPO, Candiolo, Italy

**Keywords:** nanomedicine, pancreatic cancer, nanoparticle, theranostics, biological barriers

## Abstract

Pancreatic adenocarcinoma has the worst outcome among all cancer types, with a 5-year survival rate as low as 10%. The lethal nature of this cancer is a result of its silent onset, resistance to therapies, and rapid spreading. As a result, most patients remain asymptomatic and present at diagnosis with an already infiltrating and incurable disease. The tumor microenvironment, composed of a dense stroma and of disorganized blood vessels, coupled with the dysfunctional signal pathways in tumor cells, creates a set of physical and biological barriers that make this tumor extremely hard-to-treat with traditional chemotherapy. Nanomedicine has great potential in pancreatic adenocarcinoma, because of the ability of nano-formulated drugs to overcome biological barriers and to enhance drug accumulation at the target site. Moreover, monitoring of disease progression can be achieved by combining drug delivery with imaging probes, resulting in early detection of metastatic patterns. This review describes the latest development of theranostic formulations designed to concomitantly treat and image pancreatic cancer, with a specific focus on their interaction with physical and biological barriers.

## Introduction

Pancreatic ductal adenocarcinoma (PDAC) is the fourth leading cause of cancer death in Europe and in the US (Siegel et al., 2018). The 1-year overall survival is limited to a discouraging 29%, while the overall survival at 5-year post-diagnosis is <10% (Siegel et al., 2018). The major problem is that most patients remain asymptomatic until late in their course and present at diagnosis with an already infiltrating and incurable disease (Smith et al., 2015). For the small percent of patients (19%) who present at diagnosis with local, partly resectable disease the 5-year survival reaches 27%, a prognosis that still remains dismal (Garrido-Laguna and Hidalgo, 2015).

PDAC evolves from early precursor lesions, including pancreatic intraepithelial neoplasia (PanIN), intraductal papillary mucinous neoplasms (IPMN) and mucinous cystic neoplasia (MCN), a highly invasive neosplasms characterized by an ovarian-type stroma and a mucin-producing epithelium (Hruban et al., 2007; Distler et al., 2014; Pusateri and Krishna, 2018). While PanIN often occurs as a progressive multifocal disease with hardly detectable small lesions (Bardeesy and DePinho, 2002; Makohon-Moore and Iacobuzio-Donahue, 2016), IPMNs mostly localize in the main pancreatic duct and in its related branches (Torisu et al., 2019).

Several signaling pathways, such as RAS, PI3K, and Hedgehog (Hh) are known to play a role in supporting tumorigenesis and progression (Morris et al., 2010; Cowan and Maitra, 2014). In spite of the extensive research that led to significant improvement in the understanding of the evolution of this disease, little advancement has been made toward more efficient therapeutic and early detection options for PDAC (Matsubayashi et al., 2019). The lack of indicative clinical signs and of disease-specific biomarkers, makes early detection extremely difficult (Adiseshaiah et al., 2016). In addition, pharmacological treatments remain largely ineffective, due to the difficulty in penetrating the tumor microenvironment (Conroy et al., 2011; Zhao et al., 2018). PDAC is characterized by a dense, desmoplastic stroma consisting of different cellular and acellular components (e.g., collagen and fibrin), which impedes efficient drug delivery, generates solid stress and increases interstitial fluid pressure (IFP), resulting in blood vessels collapse and in the generation of a hypoxic tumor microenvironment (Rucki, 2014; Xie and Xie, 2015; Dougan, 2017).

Nanomedicine formulations, e.g., formulation of drugs into nano-size delivery vehicles, such as liposomes and polymer nanoparticles (NPs), represent a valuable option in PDAC treatment by virtue of their ability to overcome biological barriers, protect their payload from degradation, and to achieve targeted delivery (El-Zahaby et al., 2019). Depending on their size, shape and surface charge, NPs have been shown to passively accumulate into tumors through the enhanced permeability and retention (EPR) effect (Maeda, 2001) and to actively interact with cancer cells after surface-modification with specific ligands (Yu et al., 2009), thereby enhancing selectivity and reducing undesired side effects of chemotherapy.

Although the EPR effect is not relevant in PDAC due to blood vessel collapse and to the presence of a dense desmoplastic stroma (Tanaka and Kano, 2018), several nanomedicine-based strategies have been designed and tested for the treatment of this disease (Adiseshaiah et al., 2016; Meng and Nel, 2018). For instance, conjugation of Gemcitabine (GEM) to the natural lipid squalene (SQ-GEM) to form self-assembled nanoparticles of 130 nm in size has been shown to enhance the stability of GEM and to reduce its de-activation by cytidine deaminase (Couvreur et al., 2008). SQ-GEM significantly reduced metastatic colonization and enhanced survival of mice bearing orthotopic Panc1 pancreatic tumors, compared to equivalent doses of free GEM (Réjiba et al., 2011). Another example is the liposomal formulation of Irinotecan (MM-398), which in combination with 5-fluoruracin and leucovorin (5-FU/LV) is currently recommended as second line therapy after failure of GEM treatment (Ko et al., 2013; Wang-Gillam et al., 2019; Woo et al., 2019).

In spite of these promising results, cell intrinsic (e.g., drug resistance) and cell extrinsic (e.g., tumor microenvironment) barriers should be overcome to facilitate drug accumulation in pancreatic tumors, coupled with better diagnostic and imaging modalities (Yang et al., 2012; Meng and Nel, 2018). A new class of theranostic nanomedicines that combines imaging and therapeutic options in a single platform may address this need.

Herein, we discuss the recent advancement in the design of nanosystems to improve imaging and treatment of PDAC.

## Physical And Biological Barriers in PDAC

PDAC is characterized by a thick desmoplastic stroma, composed of several cell types (including endothelial and immune cells), embedded in a dense matrix composed of fibrin, collagen, hyaluronan, and fibronectin (Cowan and Maitra, 2014; Rucki, 2014). Neoplastic cells account for <20% of the tumor mass, while the stromal volume covers up to 70% of the total tumor volume (Yang et al., 2012).

During PDAC progression, secretion of pro-inflammatory cytokines by tumor cells stimulates extracellular matrix (ECM) deposition by fibroblasts and stellate stromal cells (Hwang et al., 2008; von Ahrens et al., 2017). The continuous generation of a dense stroma generates solid stress which, together with the collapse of the lymphatic drainage in the center of the tumor, contributes to the increased intratumoral IFP and the consequent vessel compression, reduced perfusion, and generation of a hypoxic environment (Adiseshaiah et al., 2016; Meng and Nel, 2018). As a result, approximately 80% of blood vessels in PDAC are non-functional, poorly fenestrated, and surrounded by a thick layer of pericytes, that impede efficient accumulation of nanomedicines into the tumor. Moreover, pancreatic stellate cells secrete cytokines and growth factors that generate an immune-suppressive microenvironment (Thind et al., 2017). This feature of stellate cells is further amplified during tumor progression, because cancer cells induce their differentiation in two subtypes of cancer-associated fibroblasts, respectively showing a pro-inflammatory or a pro-fibrogenic phenotype (Öhlund et al., 2017). This concept has been further reinforced by single cell transcriptome analysis (Ligorio et al., 2018) performed on human PDAC underscoring a wider fibroblast heterogeneity, which locally influences the proliferative and metastatic potential of cancer cells.

## Modulation of PDAC Microenvironment With Nanomedicine

As summarized in Table 1, several strategies have been implemented to design nanomedicines that can negotiate with the microenvironmental barriers in PDAC through alleviation of the stroma burden (Thompson et al., 2010; Provenzano et al., 2012; Bhaw-Luximon and Jhurry, 2015), normalization of tumor blood vessels, or by eliciting nanoparticle-mediated immunogenic cell death (Zhao et al., 2016), as thoroughly discussed by Adiseshaiah et al. (2016) and by Meng and Nel (2018).

**Table 1:** Nanomedicines for imaging and/or treatment of PDAC.

**Therapeutic strategy**	**Nanocarrier**	**Encapsulated agents**	**Achieved results**
Stromal depletion via hyaluronan accumulation	Pegylated hyaluronidase	Abraxane + GEM	45% Response rate 11.5 months median overall survival (Jacobetz et al., 2013)
Stromal depletion via hedgehog inhibition	Polymer NPs	Paclitaxel + cyclopamine	63% higher inhibition of tumor growth (Hingorani et al., 2018)
Stromal homeostasis via PSC reprogramming	Pegylated gold NPs	Retinoic acid + HSP47-siRNA+GEM	Suppressive effect in sub-cutaneous and orthotopic tumor model (Catenacci et al., 2015)
Reduction of hypoxic microenvironment	Polymer NPs	HIF1α-siRNA + GEM	Significant reduction of tumor size and metastasis prevention (Jaster et al., 2003)
Sensitization to radiotherapy via ROS	Cerium Oxide NPs	Cerium oxide	Significant reduction in tumor weight and volume (Zhao et al., 2015)
Targeted pH-driven gene silencing	Polymer NPs	GEM + GDC 0449	Selective internalization and enhanced intratumor accumulation (Vassie et al., 2017)
Temperature-triggered drug release	Hybrid NPs	GEM	4.4-fold decreased tumor weight and reduction of tumor size (Zeiderman et al., 2016)
Targeted intracellular hyperthermia	Gold NPs	Cetuximab or PAM4	Significant reduction of tumor size after 6 weeks of combined therapy (Ray et al., 2019)
Targeted chemotherapy + MRI	Iron oxide NPs	Doxorubicin/iron oxide NPs	66.6% Inhibition of tumor growth (Mattheolabakis et al., 2015)
Targeted Enzyme Responsive Drug Release + MRI	Iron oxide NPs	GEM/iron oxide NPs	Improved intracellular release and tumor growth inhibition up to 50% (Tummers et al., 2018)
Magnetic hyperthermia + chemotherapy + MRI	Polymer NPs	GEM/fluorescent iron oxide NPs	Significant tumor regression and MRI contrast enhancement (Hoogstins et al., 2018)
Image-guided targeted photothermal therapy	Carbon nanotubes	Cyanine 7	Dynamic disease monitoring and improved median survival time (Rosenberger et al., 2015)
Targeted intra-operative fluorescent imaging	Polymer NPs	Indocyanine green	Early detection of primary tumor and splenic metastases (Handgraaf et al., 2014)
Targeted multi-modal Imaging	Polymer NPs	Iron oxide NPs + FITC	Selective tumor accumulation and active disease monitoring (Vahrmeijer et al., 2013)

Stroma depletion through delivery of pegylated hyaluronidase (PEGPH2O) was shown to enhance accumulation of high molecular weight tracers into pancreatic tumors (Jacobetz et al., 2013). Tested in combination with Abraxane and GEM in clinical trials with patients whose tumors had high hyaluronan content, an objective response rate of 45 vs. 31% and a median overall survival of 11.5 vs. 8.5 months was achieved in comparison with Abraxane/GEM therapy (Hingorani et al., 2018). Inhibition of signal pathways involved in stroma deposition, such as Hh, was implemented to facilitate accumulation of NPs to PDAC tumor models. Zhang et al. (2016) showed that oral administration of cyclopamine, a Hh inhibitor, reduced fibronectin content and enhanced tumor vascularization, resulting in a significantly higher accumulation of NPs in subcutaneous Capan-2 xenografts. Using paclitaxel (PTX)-loaded NPs combined with cyclopamine, they achieved a 63% increased inhibition of tumor growth (Zhang et al., 2016). In spite of these results, the Hh inhibitor Vismodegib combined with GEM failed to produce significant clinical benefit to patients with metastatic PDAC. No significant improvement in the overall survival or in the disease free progression was observed in comparison to standard treatment with GEM alone (Catenacci et al., 2015).

In addition to these discouraging results, other reports have shown that stroma depletion may facilitate cell proliferation and worsen the metastatic spreading, thus reducing the potential applicability of these therapies in PDAC treatment (Kiesslich et al., 2012; Özdemir et al., 2014; Adiseshaiah et al., 2016).

As an alternative to stromal depletion, Han et al. (2018) proposed to restore the fibrotic stromal homeostasis in PDAC by reprogramming pancreatic stellate cells (PSCs). They reported on the design of pH-responsive pegylated gold nanoparticles co-loaded with all-*trans* retinoic acid (ATRA) and heat shock protein 47(HSP47)-small interfering RNA (siRNA). ATRA is involved in maintaining PSCs homeostasis and quiescence, while silencing of HSP47 has the potential to reduce collagen accumulation and, consequently, to normalize the desmoplastic stroma (Jaster et al., 2003; Masamune and Shimosegawa, 2009). Combined with GEM treatment, these particles showed significant tumor suppressive effect in both, sub-cutaneous and orthotopic, PSC/PANC-1 xenografts in mice.

Knockdown of target genes involved in drug resistance, and in tumor invasion by RNA interference, is another possible strategy to modulate PDAC microenvironment (Burnett and Rossi, 2012). NPs have demonstrated to improve the biodistribution and to reduce clearance of siRNAs and micro-RNAs (miRNAs) and have been used in combination with cytotoxic drugs, such as GEM or Doxorubicin (Zhao et al., 2015; Gibori et al., 2018; Chen et al., 2019).

As an example, inhibition of the hypoxia inducible transcription factor HIF1α through siRNA combined with GEM release was proposed by Zhao et al. (2015). The hypoxic microenvironment in PDAC is responsible for the activation of genes that regulate invasion, angiogenesis, resistance to treatment and proliferation, driven mostly by the secretion of HIFs (Feig et al., 2012). GEM-loaded, lipid-coated polymer NPs, where siRNA was complexed to positively charged polylysine residues on the surface of NPs, significantly delayed the growth of subcutaneous PANC-1 tumor xenografts, demonstrating a synergistic effect between HIF1α down-regulation and GEM. Moreover, the combination therapy significantly reduced tumor size in an orthotopic PDAC model, as compared to un-encapsulated siRNA and GEM, or with particles loaded with GEM only. In addition, no peritoneal metastases were observed in the group treated with the combination therapy, while all other animals had signs of liver and peritoneal secondary tumors.

Since PDAC microenvironment generates resistance to chemo and radiotherapy (RT), Wason et al. proposed the delivery of cerium oxide nanoparticles (CONPs) to modulate production of reactive oxygen species (ROS) that sensitized PDAC cells to radiotherapy (RT) (Wason et al., 2013; Vassie et al., 2017). CONPs-based pretreatment limited tumor growth in an orthotopic L3.6pl tumor model in athymic nude mice, leading to a significant reduction in tumor weight (*P* = 0.0112) and volume (*P* = 0.0006) as compared to RT alone.

## Smart Nanomedicines in PDAC Treatment

**Figure 1:**
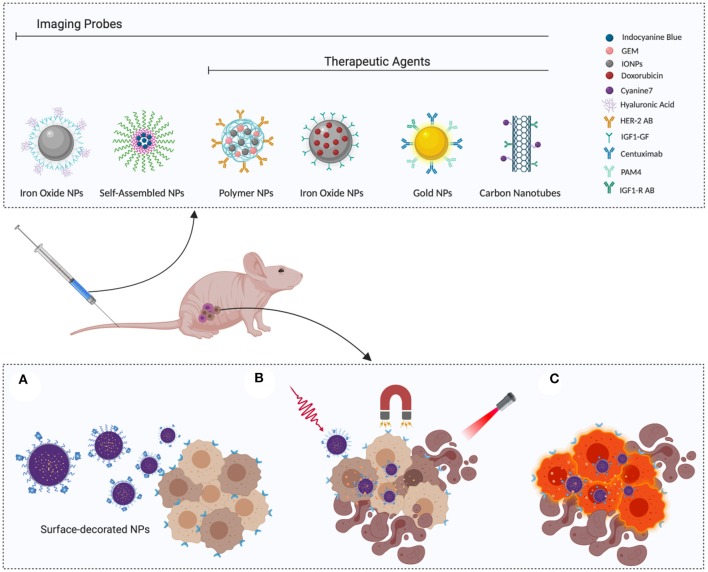
Smart nanoparticles for PDAC theranostic: **(A)** Surface-functionalized nanoparticles actively recognize tumor cells, thereby enhancing selective accumulation. **(B)** Once they reach the target site, release can be triggered by applying external stimuli, such as magnetic field or irradiation. **(C)** Selective recognition of cancer cells can be exploited to enhance their visualization, favoring complete eradication during surgery or disease monitoring with classic diagnostic tools, such as PET or MRI. Image created with Biorender.

Smart NPs are designed respond to environmental or external stimuli to trigger drug release after passive or active tumor accumulation, as schematized in Figure 1 (Zeiderman et al., 2016; Mattu et al., 2018).

Ray et al. (2019) proposed a pH-responsive platform based on block co-polymers of PEG-b-poly (carbonate) loaded with GEM and the Hh inhibitor GDC 0449. These NPs respond to the low pH of the extra- (pH 6.9–6.5) and intra-cellular compartments (pH 5.5–4.5) in PDAC, by virtue of the presence of tertiary amine side chains that promote disassembly of NPs under acidic conditions. To facilitate NPs accumulation in PDAC, the surface was modified with an iRGD peptide that selectively targets neuropilin and integrin receptors over-expressed by tumor cells. Successful accumulation was achieved and NPs were detected into BxPC-3 tumor xenografts up to 6 hours post systemic administration (Ray et al., 2019).

Temperature-triggered drug release was proposed by Oluwasanmi et al. (2017) after passive accumulation of NPs into PDAC xenografts, followed by external laser irradiation. They designed thermo-responsive hybrid NPs (HNPs) and linked GEM through a thermosensitive linker containing the Diels–Alder adducts, that are cleaved upon heat generation, thus triggering GEM release at the tumor site (Gregoritza and Brandl, 2015). When administered *in vivo* to BxPC-3 xenografts, the formulation showed enhanced anti-cancer activity, demonstrated by a 4.4-fold decreased tumor weight and reduction of tumor size when compared to GEM-loaded HNPs without laser irradiation.

Gold NPs (Au NPs) stimulated with external radio frequency (RF) irradiation have also been proposed for the non-invasive intracellular hyperthermia of PDAC (Glazer et al., 2010). Au NPs conjugated with Cetuximab or PAM4, for active targeting of epidermal growth factor receptor1 (EGFR-1) and mucine-1 (MUC-1), were intraperitoneally administered to mice bearing PANC-1 or Capan-1 xenografts. PAM4-conjugated Au NPs exhibited the highest tumor internalization. When combined with RF in the first 2 weeks of treatment, these NPs produced a significantly higher reduction of tumor size with minimal side effects, compared to unconjugated NPs or to conjugated NPs in absence of the external RF.

## Theranostic Nanoparticles

Theranostic NPs have the potential to localize imaging agents together with therapies at the tumor site (Handgraaf et al., 2014). Early detection and surgical resection have been shown to increase the mean 5-year survival of PDAC patients up to 31.7 ± 3.6 months (Cleary et al., 2004), highlighting the possibility to exploit the tumor-accumulation ability of NPs to deliver imaging agents for early recognition of PDAC (Vahrmeijer et al., 2013).

Qi et al. (2018) designed a near infrared fluorescent probe by encapsulating indocyanine green (ICG) into hyaluronic acid (HA) NPs (NanoICG). The fluorescence emission of ICG could be detected to a depth of 8 mm in tissues and was exploited to facilitate visualization of the infiltrating tumor tissue. The affinity of HA for the membrane receptor CD44 over-expressed by pancreatic cancer cells was exploited to enhance NPs accumulation into PDAC through active recognition mechanisms (Mattheolabakis et al., 2015). High tumor accumulation was achieved after administration to mice bearing a syngeneic orthotopic PDAC model. The fluorescence signal from the encapsulated ICG allowed the detection of the primary tumor as well as the splenic metastases to a much higher extent when compared to free ICG, confirming the targeting-ability of HA NPs toward PDAC.

The disease accumulation properties of NPs could be leveraged to also facilitate disease visualization during surgery (Qi et al., 2018). Recently, high-resolution fluorescent imaging agents coupled to antibodies have been used in small in-patients studies, for the detection of the primary disease or the presence of small metastatic sites during resection surgery (Hoogstins et al., 2018; Tummers et al., 2018). This may facilitate identification of the resection margins and quantification of the residual disease, albeit the clinical benefit still remains to be demonstrated.

Combination of magnetic resonance imaging (MRI) with fluorescence imaging, by co-encapsulation of superparamagnetic iron oxide NPs (IONPs) was also proposed. For instance, IONPs and fluorescein isothiocyanate (FITC) were co-encapsulated into HA NPs to exploit selective recognition of HA by CD44 receptors (Luo et al., 2019), and into NPs modified with tissue plasminogen activator-derived peptides with high affinity toward galectin-1, overexpressed by pancreatic cancer cells (Rosenberger et al., 2015). Accurate monitoring of tumor growth with MRI was achieved in both cases, after active accumulation of NPs in tumors.

MRI imaging combined with doxorubicin (Dox) chemotherapy was proposed for PDAC theranostic (Zhou et al., 2015). IONPs were conjugated to human insulin-like growth factor1 (IGF1) that selectively binds to IGF1-receptors in pancreatic cancer cells, and loaded with Dox. IGF1-IONPs exhibited excellent tumor penetration ability after IV administration in an orthotopic patient-derived tumor model. Moreover, when administered intratumorally, these particles led to a significant inhibition of tumor growth (66.6%), compared to treatment with free Dox, non-targeted IONP-Dox, or PBS. Enhanced MRI contrast was obtained for the group treated with IGF1-IONP-Dox, while no significant contrast was observed in non-targeted IONP-Dox, suggesting IGF1R-mediated accumulation.

Lee et al. (2013) designed urokinase plasminogen activator (uPAR)-modified IONPs loaded with GEM via an enzyme-cleavable tetrapeptide linker. They achieved improved endocytosis through active recognition of uPAR receptors, and a consequently higher intracellular release of GEM and MRI contrast. Moreover, inhibition of tumor growth (up to 50%) was obtained in an orthotopic pancreatic cancer model.

IONPs have the potential to generate heat after external irradiation (Jaidev et al., 2017). Jaidev et al. (2017) developed polymeric NPs for MRI, magnetic hyperthermia (MHT) and chemotherapy for application in PDAC. Poly(lactide-co-glycolide) (PLGA)-based NPs encapsulating fluorescent IONPs and GEM were conjugated with anti-human epidermal growth factor receptor 2 (HER-2) antibody. When administered in subcutaneous MIAPaCa-2 tumor models in combination with mild hyperthermia, NPs led to a significant tumor regression; moreover, a remarkable contrast enhancement was observed in T2-MRI images of treated mice.

Single-walled carbon nanotubes (SWNTs) can also convert heat after near infrared (NIR) irradiation, resulting in localized hyperthermia that leads to tumor cells death via ROS production (Singh and Torti, 2013). Lu et al. (2019) formulated anti-IGF-1R antibody functionalized SWNTs for enhanced imaging-guided cytotoxic photothermal therapy (PTT) of PDAC. SWNTs exhibited preferential accumulation into tumors, resulting in dynamic monitoring of the disease. Fluorescence-guided PTT significantly improved the survival of mice bearing an orthotopic PDAC model, compared to groups treated with PBS or only with NIR laser.

## Conclusions And Future Directions

PDAC remains an incurable disease. The dense stroma, the lack of vascular access, and the heterogeneous microenvironment, make PDAC extremely refractory to treatment penetration, requiring the design of smart strategies to by-pass these barriers and to maximize treatment accumulation in the tumor (Gibori et al., 2018).

Late disease detection worsens patient outcome, making surgical resection ineffective. The tumor-accumulation and targeting ability of nanomedicines could be leveraged to improve disease detection at early stage, considerably improving survival and enhancing the extent of surgical resection (Handgraaf et al., 2014). Early stage detection may also result in more efficacious nanomedicine-based treatments, for instance coupled with stroma-depleting agents which would further potentiate disease homing.

Local delivery is an attractive, yet poorly exploited, alternative to treat PDAC. Local administration avoids the stroma protection and overcomes the restricted vascular access, potentially reducing side effects, as demonstrated by the encouraging results of the siG12D-LODER implant (Adiseshaiah et al., 2016). SiG12D-LODER is a biodegradable implant for the local delivery of liposomal-encapsulated anti-KRAS siRNA that is placed near the tumor by means of standard endoscopic surgery (Golan et al., 2015). In a small subset of PDAC patients, stabilization of tumor growth, and partial response was achieved in combination with chemotherapy, suggesting the potential of this smart delivery method (Schultheis et al., 2014).

As discussed above, extensive research has shown the potential of nanomedicine in PDAC and some formulations, such as albumin-bound paclitaxel (Abraxane) and liposomal irinotecan (MM-398), reached clinical approval (Kalra et al., 2014; Goldstein et al., 2015; von Ahrens et al., 2017). It must be noted that although MM-398 in combination with other cytotoxic agents improved patient survival, it failed to produce similar improvements when used as mono-therapy (Adiseshaiah et al., 2016; Kipps et al., 2017; Wang-Gillam et al., 2019).

Additionally, the different animal models, cell source, tumor location (e.g., heterotopic vs. orthotopic), and nanoparticle design used in pre-clinical research may result in difficult comparison between published research and in the overestimation of the results (Murtaugh, 2014; Adiseshaiah et al., 2016; Leong et al., 2019).

Efforts toward standardization of research and treatment protocols may further improve the potential of nanomedicine in the field.

## Author Contributions

All authors listed have made a substantial, direct and intellectual contribution to the work, and approved it for publication.

### Conflict of Interest

The authors declare that the research was conducted in the absence of any commercial or financial relationships that could be construed as a potential conflict of interest.
